# Re-appraising the evidence for the source, regulation and function of p53-family isoforms

**DOI:** 10.1093/nar/gkae855

**Published:** 2024-10-15

**Authors:** Ignacio López, Irene Larghero Valdivia, Borivoj Vojtesek, Robin Fåhraeus, Philip J Coates

**Affiliations:** Biochemistry, Faculty of Science, Universidad de la República, Iguá 4225, Montevideo 11400, Uruguay; Cell Biology Unit, Institut Pasteur de Montevideo, Mataojo 2020, Montevideo 11400, Uruguay; Biochemistry, Faculty of Science, Universidad de la República, Iguá 4225, Montevideo 11400, Uruguay; RECAMO, Masaryk Memorial Cancer Institute, Zluty kopec 7, Brno 65653, Czech Republic; RECAMO, Masaryk Memorial Cancer Institute, Zluty kopec 7, Brno 65653, Czech Republic; Inserm UMRS 1131, Institut de Génétique Moléculaire, Université de Paris Cité, 27 rue Juliette Dodu, Hôpital St. Louis, Paris F-75010, France; Department of Medical Biosciences, Building 6M, Umeå University, Umeå 90185, Sweden; RECAMO, Masaryk Memorial Cancer Institute, Zluty kopec 7, Brno 65653, Czech Republic

## Abstract

The p53 family of proteins evolved from a common ancestor into three separate genes encoding proteins that act as transcription factors with distinct cellular roles. Isoforms of each member that lack specific regions or domains are suggested to result from alternative transcription start sites, alternative splicing or alternative translation initiation, and have the potential to exponentially increase the functional repertoire of each gene. However, evidence supporting the presence of individual protein variants at functional levels is often limited and is inferred by mRNA detection using highly sensitive amplification techniques. We provide a critical appraisal of the current evidence for the origins, expression, functions and regulation of p53-family isoforms. We conclude that despite the wealth of publications, several putative isoforms remain poorly established. Future research with improved technical approaches and the generation of isoform-specific protein detection reagents is required to establish the physiological relevance of p53-family isoforms in health and disease. In addition, our analyses suggest that p53-family variants evolved partly through convergent rather than divergent evolution from the ancestral gene.

## Graphical Abstract

**Graphical Abstract:**
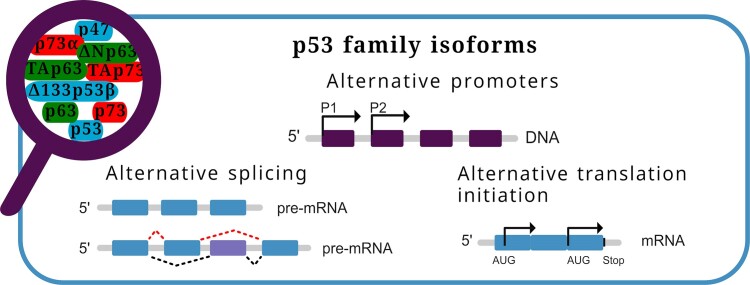

## Introduction

The members of the p53 protein family of transcription factors are encoded by three evolutionarily related genes: *TP53*, *TP63* and *TP73*. Each gene is proposed to produce a number of different isoforms, employing different promoters, differential exon–exon splicing and/or alternative initiation of translation. The production of protein variants lacking certain domains and able to form hetero- and homo-oligomers provides a powerful example of how the functional repertoire of a gene can be increased exponentially. In general, p53-family isoforms can be categorized from four aspects: mechanisms generating isoforms, alteration of functional domains, regulation of isoform expression and biological function.

Alternative splicing is a major mechanism for producing protein isoforms. It has been estimated that more than 90% of human genes undergo alternative splicing, with the less abundant variants representing 15% or more of the total transcripts for that gene in 80% (1,2). Remarkably, the number of alternatively spliced transcripts is estimated to be 10-fold higher than the number of transcribed genes, particularly in tumours and rapidly growing cell lines. However, the role of alternative splicing as the major driver of proteome complexity is questionable (3). Within the alternative splicing landscape, it must be borne in mind that transcripts present at low levels may arise from altered RNA polymerase fidelity combined with imprecise transcription initiation and faulty splicing machinery, rather than representing carefully controlled processes. RNA polymerase II may initiate at minor transcriptional start sites in 95% of cases (4) and the splicing machinery has error rates estimated as 0.7% per intron–exon junction (5) to ≥ 1 mistake per 10^5^ splicing events (6). Thus, 90% or more alternative transcripts derived from moderately abundant mRNAs may be due to errors (4,7). Moreover, definitive experimental evidence is lacking for up to 95% of curated functionally distinct splice isoforms, and only 13% have moderate levels of evidence for their existence (8). On the other hand, recent in-depth proteomics have confirmed the presence of most proteins produced from frame-preserving alternative splicing (9), and such approaches will lead to a clearer picture of the extant proteome derived from alternative transcripts in the future. Thus, although widely accepted, the functional authenticity of many predicted alternative transcripts remains contentious, particularly for low abundance variants expressed in combination with high levels of the major product. Of particular relevance for p53-family isoforms, studies often employ highly sensitive amplification methods that are able to detect a single mRNA molecule of an individual isoform within an entire sample, increasing the risk of detecting transcripts resulting from errors rather than identifying physiologically relevant isoforms. It should also be kept in mind that population-level nucleic acid-based studies provide average mRNA levels, hiding intercellular variations linked to tissue and cell heterogeneity. Moreover, they do not perform well for low-abundant targets that include transcription factors, nor for identifying alternative splicing events (10). These two aspects can, to some extent, be tackled by single-cell RNA-sequencing (scRNA-seq) approaches, keeping in mind that scRNA-seq studies generally do not report isoform levels and can be inaccurate due to short read lengths and numbers of low-abundant transcripts. In this respect, although not as sensitive as polymerase chain reaction (PCR)-based methods, Northern blotting is an ideal method for identifying and directly quantifying the relative levels of mRNA isoforms, but is rarely, if ever, performed these days.

A second general mechanism for producing protein variants is the use of alternative promoters for transcription initiation. Even though it remains controversial, there is good evidence that alternative transcription start and termination sites are responsible for generating most isoforms in human tissues (11). In particular, promoter usage often dictates the presence or absence of initiating AUG codons in the 5′ regions of variant mRNAs, leading to different N-terminal sequences in the translated protein product. Importantly, the decision of which promoter is employed is dependent on the presence/absence of specific transcription factors and epigenetic marks such as histone acetylation and DNA methylation, providing simple mechanisms to control which promoter is used in any given cell at any given time. Thus, alternative promoter usage is tightly regulated during cell state transitions and mammalian cell development (12–15), and can itself affect downstream splicing and 3′ end selection (4,16).

A third mechanism to generate protein isoforms is alternative initiation of mRNA translation (17,18). Unlike alternative transcripts, which are easily detected through mRNA sequencing or reverse transcriptase-PCR (RT-PCR)-based techniques, alternative translation initiation products are derived from the same transcript and therefore require detection at the protein level. Thus, strategies for their identification are based mainly on protein size differences combined with the use of antibodies that recognize epitopes shared and not shared among the variants. Such approaches rely on the availability of antibodies with known epitopes in the N-terminus and elsewhere, which are not always available (or the precise epitopes of commercial antibodies are not provided). In addition, apparent molecular weight in gel electrophoresis changes according to post-translational modifications (PTMs) and proteolysis, making it a non-trivial task to definitively identify alternative translation products derived from the non-canonical AUG. Mutagenesis approaches directed to putative AUG initiation codons to eliminate one isoform can be used to determine whether mRNAs are subject to alternative initiation, but this is only easily implemented for expression of cDNA constructs in cell lines. However, non-AUG codons may initiate translation. For example, CUG-mediated initiation upstream of the canonical annotated AUG translation start site generates a longer c-Myc variant protein (18). As more studies demonstrate alternative translation initiation as a source of protein isoforms under different cellular conditions, their contribution to protein diversity will become better known.

A fourth general mechanism for producing protein variants is through proteolytic cleavage of precursor proteins. There are many such examples, including protease activation of hormones and receptors [reviewed in (19,20)], but there is little evidence that regulated proteolytic cleavage is a source for p53-family isoforms and this mechanism will therefore not be discussed further.

In the following sections, we discuss the evidence for the presence of isoforms produced from each of the three p53-family genes, including their relative levels and the mechanism(s) involved in their production and regulation, as well as their functions. We also provide suggestions for methodological approaches to improve the confidence for their potential biological relevance. Despite concentrating on the functions and relevance of the encoded protein variants, we recognize that mRNAs may exert non-coding functions as scaffolding entities for RNA binding proteins (21–24), and that aggregation (25–27) may potentiate p53-family isoform effects, although this propensity has been questioned recently (28). These are areas that will require further investigation. Whilst we focus here on the p53-family, these considerations are equally valid for investigating all purported gene/protein variants. Given the vast literature on p53-family isoforms, it is not possible to include all of the original articles and we apologize to authors that are not cited due to space constraints.

### TP63

**Figure 1.**
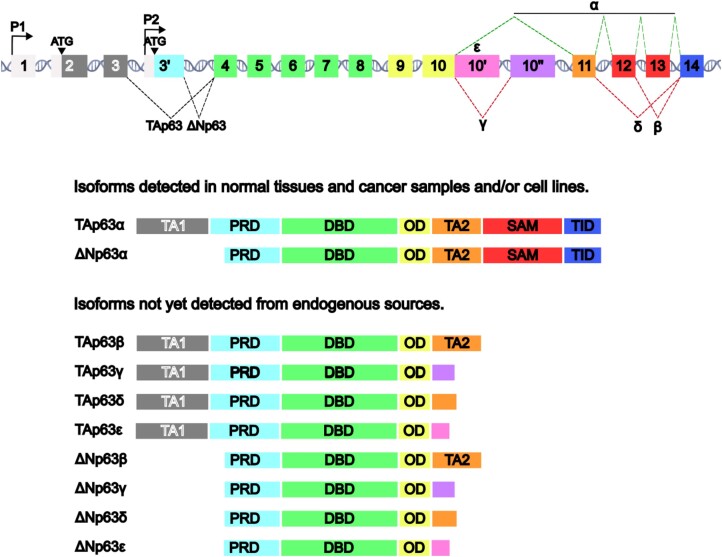
*p63 protein isoforms*. **(Top)** Gene architecture of the human *TP63* locus. Boxes represent exons and DNA cartoon represents introns. Exons are numbered according to canonical annotation and cryptic exons are named 3′, 10′ and 10″. Promoters P1 and P2 driving production of TAp63 and ΔNp63, respectively, are represented by arrows. Translation initiation codons (ATG) are indicated with arrowheads. Splicing patterns are signalled by dashed lines that connect the regions involved. Dashed lines at the 5' end (on the left) refer to splicing resulting from alternative promoter usage. For 3′ variants (on the right), top dashed lines are canonical (α variants) while bottom are alternative splicing patterns (β, γ, δ and *ϵ*). Shaded areas denote nucleotide sequences that correspond to different protein domains presented on the panel below. **(Bottom)** Putative p63 protein isoforms listed according to detection confidence from endogenous sources and showing the domains present. TA1 and TA2: transactivation domain. PRD, proline-rich domain; DBD, DNA-binding domain; OD, oligomerization domain; SAM, sterile alpha motif domain; TID, transactivation inhibitory domain. The unique peptides are shown for γ and ϵ. Sizes of exons and protein domains are to scale. Protein isoforms are grouped according to the evidence for their expression.

Discovered almost 20 years after p53, the *TP63* gene is the ancestral member of the family from which p73 and p53 subsequently evolved by gene duplication events (29,30). *TP63* was first identified as a cDNA sequence from rat tongue epithelium with extensive homology to *TP53* (31), and was further characterized and named a year later (32). It was realized at that early time that *TP63* produces multiple mRNA and protein isoforms, using alternative promoters to produce N-terminal variants (TAp63 and ΔNp63) and alternative splicing to produce C-terminal protein isoforms (α, β and γ). Since then, additional C-terminal variants have been described, each of which may theoretically be present as TAp63 or ΔNp63 forms (Figure 1). Indeed, up to 12 protein isoforms are currently listed in Uniprot (https://www.uniprot.org/; Q9H3D4) and 14 transcripts in Ensembl (https://www.ensembl.org/index.html Gene: *TP63* ENSG00000073282).

It is important to note from the start that there is not even a single transcript to support two of these putative isoforms (Transcript Support Level 5) and that the evidence for one other isoform is limited to a single EST (Transcript Support Level 3) in Ensembl (Supplementary Table S1). However, despite the relative lack of evidence supporting some isoforms, these transcripts and their predicted proteins are presented and annotated in respected authoritative gene collections, suggesting their functional reality and importance.

### Alternative splicing of TP63

**Figure 2.**
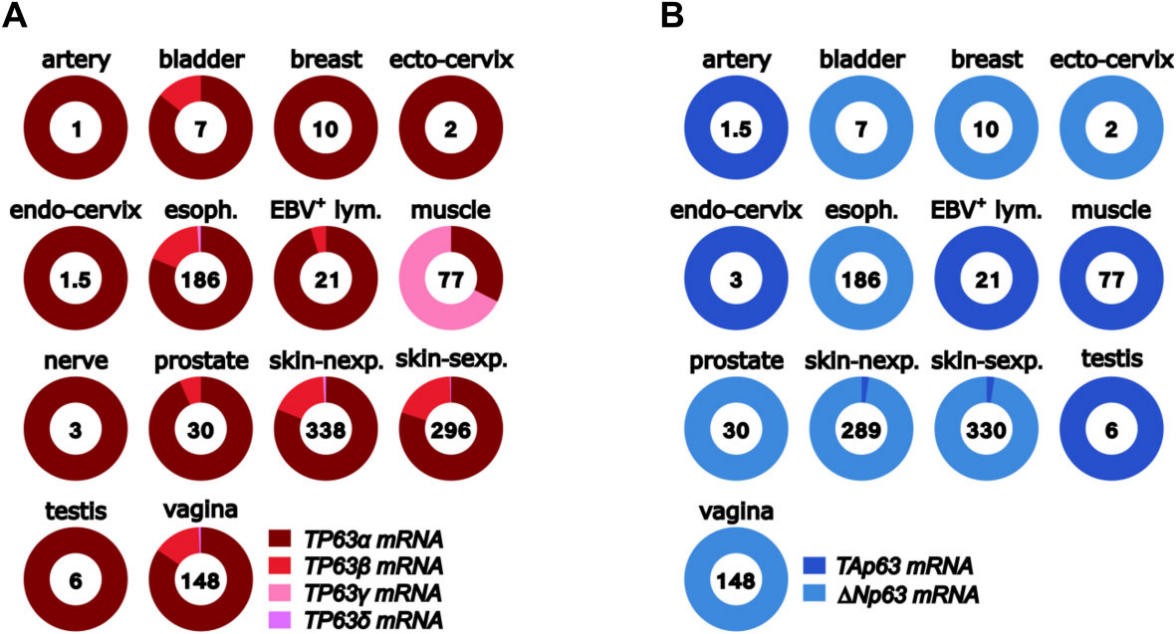
*The distribution of TP63 transcripts in human tissues*. (**A)** Proportions of alternatively spliced *TP63* mRNAs. Numbers inside the circles refer to the average number of reads for each tissue, and the shading of the circles represent the percentage of reads across 3′ exon–exon junctions that define the alternative splicing event. Data are normalized according to the number of reads of non-spliced exon–exon junctions using the approach described in (33). (**B)** Proportions of *TAP63* and Δ*NP63* mRNAs derived from alternative promoter usage. Note that absolute levels vary considerably among tissues. Data are taken from GTEx. Esoph., esophagus; EBV^+^ lym., Epstein–Barr virus-positive lymphocytes; skin-nexp., skin not exposed to sun; skin-sexp., skin exposed to sun.

Up to five p63 isoforms are proposed to arise from alternative splicing of *TP63* 3′ exons (designated α, β, γ, δ and ϵ), each of which may theoretically exist as either TAp63 or ΔNp63 protein variants (Figure 1 and Supplementary Table S1). Previous studies indicated that *TP63α* mRNA forms (*TAP63α* and *ΔNP63α*) predominate in normal tissues and tumours, and other isoforms represent only a fraction of the overall *TP63* mRNA (33–35). We analysed exon–exon junction reads that define the 3′ splice variants using the current RNA-seq data in the Genotype-Tissue Expression (GTEx) project portal containing multiple samples of normal adult human tissues (GTEx Analysis Release V8, dbGaP Accession phs000424.v8.p2 accessed on 01/02/24 and 10/08/24) (36). This analysis confirms that *TP63α* mRNA is the most common 3′ variant, with the exception of muscle, where *TP63γ* is seen along with *TP63α* (Figure 2A). *TP63β* is present at about 20% or lower total *TP63* mRNA levels in some tissues, with *TP63α* accounting for the remainder. Apart from muscle, *TP63γ* represents < 1% of total *TP63* mRNA levels (and only in the oesophagus and skin). No tissue shows levels of *TP63δ* above 1% of total *TP63* mRNA, and there is no evidence for the existence of *TP63ϵ* mRNA in any normal adult human tissue. Similar results are seen using RNA-seq in a range of cancer cells, showing *TP63α* mRNA to be the predominant form in all samples, with much lower levels of *TP63β* or *TP63γ* occasionally detected and no evidence for *TP63δ* or *TP63ϵ* (35,37).

These data contrast with studies showing the presence of 3′ alternatively spliced isoforms, often reported to show altered levels in tumours compared to normal tissue counterpart controls. This apparent discrepancy can be explained by the use of assays that target a specific *TP63* mRNA splice variant, often involving multiple cycles and/or nested PCR, sometimes followed by Southern blotting to detect the amplified product [e.g. (38)]. Although this has been, and continues to be, a common approach for investigating *TP63*, *TP53* and *TP73* mRNA isoforms, these PCR-based techniques are hyper-sensitive and, in consequence, capable of identifying just a few mRNA molecules within the sample analysed. In addition, results are presented as fold-change in the individual isoform, not as total amount of isoform compared to total amount of *TP63* mRNA. Therefore, such techniques do not indicate the functional importance of an individual isoform in tumourigenesis, particularly if the mRNA variant is swamped by far higher levels of another isoform. It must also be remembered that a low level of a particular mRNA isoform may be due to either a low number of transcripts in each cell (unlikely to have biological relevance and possibly due to erroneous rather than regulated splicing), or may indicate that only a small population of cells transcribe that specific mRNA isoform (likely to have functional effects and suggesting specific regulation). Thus, spatial single cell determination is critical for evaluating isoforms.

### Alternative promoters in TP63

In contrast to the weak evidence for some proposed alternative splicing of 3′ exons, there is overwhelming data to support the two N-terminal p63 variants, TAp63 and ΔNp63, that are derived from alternative promoters and contain distinct N-terminal amino acid sequences (Figure 1 and Supplementary Table S1). The *TAP63* and *ΔNP63* 5′ mRNAs show robust levels by standard RT-qPCR, RNA-seq and scRNA-seq, and are expressed in cell-type dependent manners (Figure 2B).

There is no evidence for alternative initiation of translation as a mechanism for producing p63 protein variants, unlike p53 and p73 (see below).

### Evidence for p63 isoform proteins

**Figure 3.**
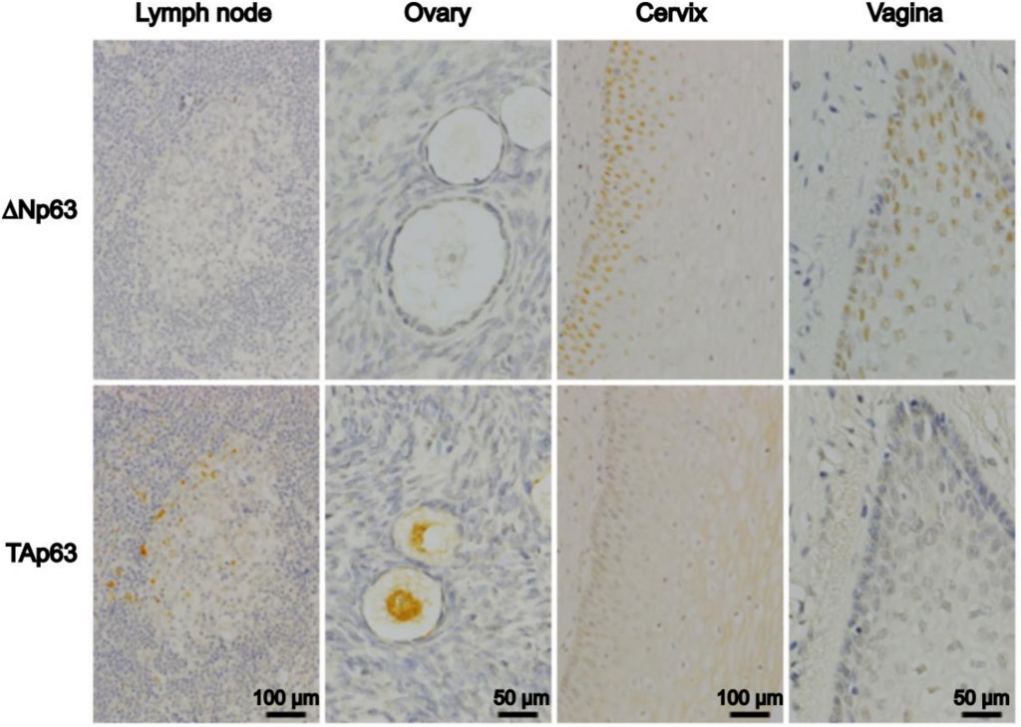
*Distribution of TAp63 and ΔNp63 protein isoforms in normal human tissues*. Paraffin sections (4 μm) of the indicated normal human tissues were immunostained for ΔNp63 (mouse monoclonal ΔNp63-1.1) or TAp63 (mouse monoclonal TAp63-4.1) (40). Antibody binding was detected using peroxidase polymer-conjugated anti-mouse immunoglobulin and 3,3′-diaminobenzidine (DAB, brown) as chromogen. Nuclei were counterstained with haematoxylin (blue).

The use of pan-p63 antibodies (that recognize all variants equally) has identified endogenous proteins in normal and tumour cells with sizes that correspond to TAp63α or ΔNp63α proteins produced by *in vitro* translation or cell transfection with *TAP63α* or *ΔNP63α* coding sequences. These proteins also show differential expression in different cells/tissues that corresponds with mRNA data (32,35). Polyclonal and monoclonal antibodies have also been produced to the unique TAp63 and ΔNp63 peptide sequences and these confirm cell- and tissue-dependent N-terminal p63 isoforms by Western blotting and at the single-cell level using immunohistochemistry (Figure 3) (35,39–41). An important side issue in detecting p53-family protein isoforms is antibody cross-reactivity, where many pan-p63 antibodies also identify p73 and/or p53 (40), and many p73 antibodies similarly show cross-reaction with p63 and/or show non-specific protein binding (42). Given that isoforms in the p53-family show overlapping molecular sizes, the use of pan-p63 or pan-p73 antibodies may therefore identify a different family member rather than a different isoform. For these reasons, antibodies to p53-family proteins require rigorous characterization to demonstrate specificity, including precise epitope mapping and direct assessment of cross-reactivity with other members of the family. Although this has been achieved for a minority of reagents (40,42), such extensive characterization is not available for commercial antibodies used in the majority of studies.

To date, there are no antibodies available to p63 C-terminal variant proteins other than p63α (35,41), which calls for the development of new tools to assess the expression patterns of these other protein variants.

### Regulation of p63 isoform mRNAs and protein activities

If different protein isoforms are produced to achieve specific functions, it follows that the levels of each isoform should be tightly regulated. Thus, the ability to influence the levels and activities of each isoform in different cell types and/or under different circumstances is important evidence for physiological relevance. For p63, cell-type specific expression of N-terminal variants is clear (Figures 2 and 3), as is their functional relevance, with TAp63 derived from P1 exhibiting p53-like properties and being an essential component of germ cell apoptosis by post-translational activation after genotoxic stress (43). In contrast, ΔNp63, derived from P2, is a lineage determinant factor for epithelial cells, involved in stem cell maintenance, growth and survival, and is overexpressed in specific cancers, sometimes associated with gene amplification (44–46).

The two *TP63* gene promoter sequences contain binding sites for different transcription factors, providing a simple method for controlling individual promoter activity depending on the presence or absence of these transcription factors in different cell types and/or under different conditions [reviewed in (44)]. DNA methylation has also been shown to differ at the two promoter regions in different cell types, and the degree of methylation inversely associates with the level of transcription from each promoter (47). In addition, numerous growth factors and signalling pathways increase or repress N-terminal *TP63* isoform mRNA and protein levels, including effects on mRNA translation and protein stability (44,47,48). p63 levels are also regulated by ubiquitin-mediated degradation pathways (44,49). Hence, the mechanisms for controlling N-terminal p63 isoforms are well-established and the complexity of regulatory factors is in keeping with the requirement for tight control of these two distinct isoform types.

In contrast, there is no firm evidence for controlled alternative splicing of 3′ exons, although the presence of high levels of *TP63γ* mRNA in muscle but not in other cells (Figure 2A) suggests cell-type-specific control of this splicing event. In addition, ΔNp63β protein levels are lower than ΔNp63α in cells transfected with these isoform cDNAs (50,51), suggesting different rates of protein synthesis or stability. There is also evidence that p63β and p63γ have higher transactivation properties than p63α due to their lack of the p63α inhibitory domain (50,51), making it difficult to ascertain the importance of these isoforms.

In summary, there is good evidence for the sources and functions of TAp63 and ΔNp63 in normal tissues and tumour cells, and their mechanisms of regulation are at least partly known. For the proposed C-terminal mRNA variants, *TP63α* is the predominant isoform in all normal tissues and tumour cells examined, except for *TP63γ* in striated muscle, which expresses *TAP63* but not Δ*NP63* mRNA. *TP63β* mRNA is detected at low-moderate levels alongside *TP63α* in epithelial tissues and tumours that express Δ*NP63* mRNA, and in lymphocytes that express *TAP63*. Thus, TAp63α and ΔNp63α are supported at the mRNA and protein levels, while ΔNp63β, TAp63β and TAp63γ are supported at only the mRNA level. Robust evidence is lacking for ΔNp63γ, and for p63δ or p63ϵ as either ΔNp63 or TAp63 forms.

### TP53

**Figure 4.**
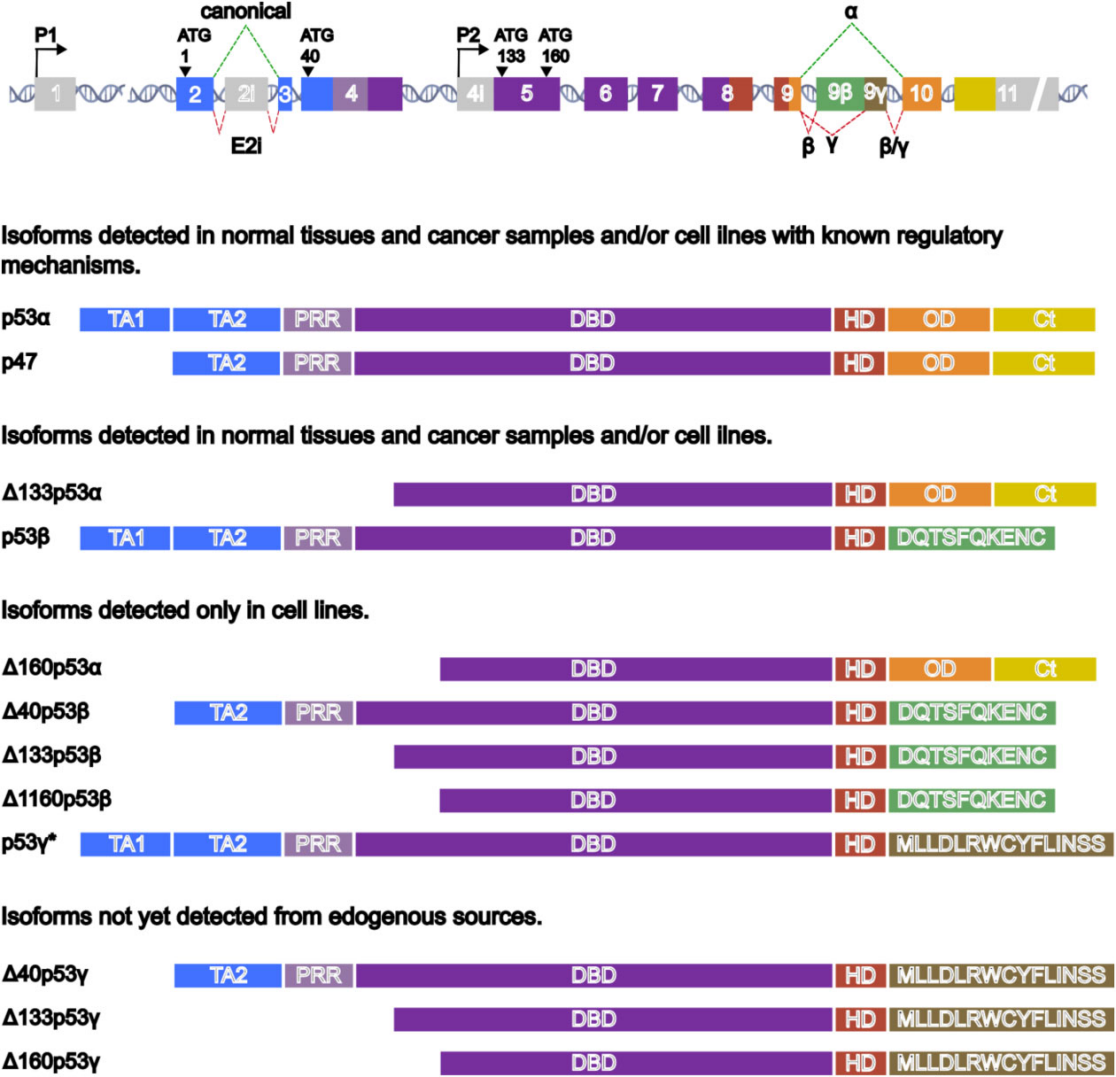
*p53 protein isoforms*.**(Top)** Gene architecture of the human TP53 locus. Boxes represent canonical and alternative exons and DNA cartoon represents introns. Exons are numbered according to canonical annotation and cryptic exons are named 2i, 9β and 9γ. Promoters P1 and P2 are represented by arrows and translation initiation codons (ATG) by arrowheads along with the position on the coding sequence of full-length p53. Splicing patterns are annotated by dashed lines that connect the regions involved. Top are canonical while bottom are alternative splicing patterns. Shaded areas denote nucleotide sequences that correspond to different protein domains presented on the panel below. **(Bottom)** Putative p53 isoforms listed according to detection confidence from endogenous sources and showing the domains present. TA1 and TA2: transactivation domain. PRR, proline-rich region; DBD, DNA-binding domain; HD, hinge domain; OD, oligomerization domain; C-t, regulatory C-terminal domain. The unique peptide sequences are shown for 9β and 9γ. Sizes of exons and protein domains are to scale, except for exon 11 and β and γ peptides. Protein isoforms are grouped according to the evidence supporting their expression from endogenous sources in different cell types and their regulation. *Endogenous expression of p53γ was inferred once in cancer cell lines under conditions affecting the activity of splicing factors, and with a complex detection approach based on specific siRNAs to alter the expression pattern of p53 isoforms combined with three Western blotting experiments: presence of signal at an expected position (∼47–48 KDa) using antibodies recognizing several p53 isoforms and absence of signal using both N-terminal and β-specific antibodies to discard p53/47 (Δ40p53α) and p53β, respectively (60).

*TP53* (*Trp53* in mouse) is one of the most highly studied genes in the human genome (52). Its product, p53, is a pivotal tumour suppressor whose activity is disrupted by mutation in more than one-third of all human cancers according to the Catalogue Of Somatic Mutations In Cancer (53). The human *TP53* gene is located on chromosome 17p13 and contains 11 canonical exons. The N-terminal region of the full-length canonical protein (also known as TAp53 or p53α but here termed p53 for simplicity) harbours the transactivation domains (TA1 and TA2) and the highly conserved Box I domain to which the E3 ubiquitin ligase MDM2 binds. TA1 and TA2 are followed by a proline-rich region (PRR) and the DNA-binding domain (DBD), the latter containing the majority of cancer-associated mutations. Further towards the C-terminus are the oligomerization domain (OD) and regulatory domains (Figure 4).

p53 constitutes a crucial hub that controls cellular homeostasis and contributes to the resolution of endogenous and exogenous stresses, including DNA damage, oncogene activation, ribosomal stress and hypoxia. To achieve the appropriate response, among which DNA repair, growth arrest, apoptosis and senescence are the most iconic, p53 has multifaceted and carefully controlled activities [reviewed in (54–56)]. How p53 integrates cellular signals to trigger such a diverse set of outputs relies on the combination of two main characteristics; (i) a diverse pattern of PTMs and (ii) a large interactome (controlled through distinct PTM patterns) with other proteins and with DNA and RNA (55,57,58). The proposed existence of p53 isoforms adds to the potential complexity through which p53 response pathways may be controlled. Twelve putative protein isoforms have been inferred (59) (Figure 4), and nine of these are currently listed in Uniprot (https://www.uniprot.org/; P04637). However, several transcripts listed in Ensembl (Gene: TP53 ENSG00000141510) have low transcript support levels, suggesting that the number of authentic variant mRNAs is lower than the predicted figure (Supplementary Table S2). Three mechanisms are reported to produce p53 protein isoforms: alternative promoters, alternative splicing and alternative translation initiation (Figure 4).

### Alternative splicing of TP53

**Figure 5.**
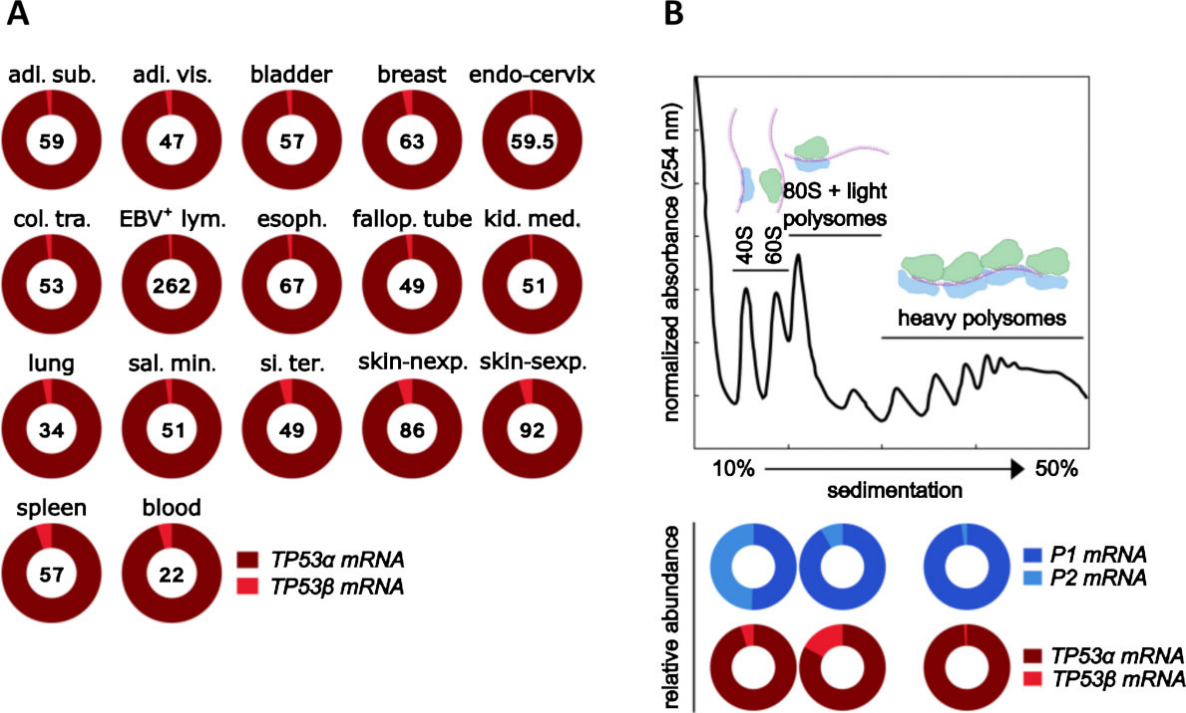
*TP53 transcripts in human tissues and their relative polysome distribution*. (**A)** Levels of alternatively spliced C-terminal *TP53* mRNAs. Numbers inside the circles refer to the average number of reads for each tissue, and the shading of the circles represent the percentage of reads across 3′ exon–exon junctions that define the alternative splicing event. Data are normalized according to the number of reads of non-alternatively spliced exon–exon junctions using the approach described in (33). The tissues shown are those for which more than one mRNA variant is present. Tissues not shown contain only *TP53α* mRNA. Data are taken from GTEx. adi. sub., adipose-subcutaneous; adi. vis., adipose-visceral; col. tra., colon-transverse; EBV^+^ lym., Epstein–Barr virus-positive lymphocytes; esoph., esophagus; fallop. tube, fallopian tube; kid. med., kidney-medulla; sal. min., salivary gland-minor; si. ter., small intestine-terminal ileum; skin-nexp., skin not exposed to sun; skin-sexp., skin exposed to sun. (**B)** Quantification of *TP53* mRNAs associated with ribosomal fractions (ribosome profiling) of HEK293 cells. Translation was halted with cycloheximide, cell extracts were generated and ribosomal fractions were isolated according to their sedimentation index on a continuous 10–50% sucrose gradient. Total RNA was precipitated, purified and used to create a library by polyA capture and reverse transcription (66,67). Samples were sequenced using Illumina PE150 technology. The figure shows a schematic representation of polysome fractionation (top) and the relative quantification of *P1* versus *P2* and α versus β *TP53* mRNAs (bottom). Concentration of ribosomal fractions was monitored by absorbance at 254 nm.

As with *TP63*, alternative splicing of 3′ exons in *TP53* mRNA is reported to result in mRNA isoforms that encode proteins with different C-terminal sequences (*α, β* and *γ*) (59,61) (Figure 4). The *TP53α* transcript arises when exons 9 and 10 are spliced together, and this is the most abundant transcript detected in all normal adult human tissues catalogued in GTEx (Figure 5A). Retention of cryptic exons 9β and 9γ within intron 9 gives rise to *TP53β* and *TP53γ* mRNAs that produce proteins with unique 10 (DQTSFQKENC) and 15 (MLLDLRWCYFLINSS) amino acid C-terminal sequences, respectively. *TP53β* mRNA is detected in 16 of the 54 normal adult tissues in GTEx, but with at least 20-fold less abundance than *TP53α* mRNA (Figure 5A) and the exon 9–9γ junction that defines *TP53γ* is not observed in GTEx. However, these data remain incomplete and specific cellular conditions not currently included may affect the relative expression of different *TP53* transcripts in specific cells or tissues. On the other hand, similar to cancer-associated *TP53* nonsense or frame-shift mutations that would produce truncated proteins, there is good evidence that these mRNAs are rapidly degraded through nonsense-mediated RNA decay (62,63).

Alternative splicing was also reported to occur within the 5′ region of *TP53*, where retention of intron 2 produces a transcript containing three stop codons in frame with the first AUG from which translation of full-length p53 begins (64,65) (Figure 4). However, there is no evidence for intron 2-retaining transcripts in GTEx. In the original study, the main *TP53* transcript was detected after 20 PCR cycles, whereas the 5′ spliced variant required 40 cycles (65). Assuming that the primers used to detect the two transcripts have similar properties, this alternative transcript would be 2^20^-fold (10^6^) less abundant than the canonically spliced *TP53* mRNA, questioning whether this would be sufficient to influence p53 activity.

It has also been shown that p53 mutations may alter p53 isoform levels: most *TP53* alternative splicing events associate with p53 frame-shift mutations (68) and transfection of common mis-sense mutant p53 increased the level of Δ160p53 (see below) (69), although this still represented only 10% of total p53 in transfected cells and much less in cancer cells that endogenously express the same mutation. Importantly, although *TP53* mRNA is detected robustly in cancer cells using RNA-seq, alternatively spliced *TP53* mRNAs are not detected without using PCR amplification (25). Similarly, isoform mRNAs represent only a small proportion of total *TP53* mRNA in tumour samples in TCGA and in cancer cell lines in the cancer cell line encyclopaedia (68).

Regardless of giving rise to biologically significant levels of protein in normal tissues or not, the levels of *TP53β* and *TP53γ* transcripts are reported to increase in some cancers, and may act as prognostic markers associated with therapeutic response and/or prognosis [reviewed in (27,70,71)]. Equally important to the issue of p53 isoforms and prognosis is to consider whether the relationship is causal or simply correlative, an often understudied issue in cancer research (72). Given that cancers show aberrant splicing of thousands of transcripts (73) that associate with tumour biology and therapeutic response (74), the clinical associations of *TP53* isoform mRNA changes are equally compatible with isoforms representing a non-specific marker of global splicing aberration rates in a tumour, rather than indicating a functional effect of the particular *TP53* isoform. Strategies to address this question are discussed in the final sections of this review. In addition, showing a causal relationship of p53 isoforms with prognosis/therapy response will require at least demonstration of necessity and sufficiency (72).

### Alternative promoters in TP53

The canonical *TP53* promoter (P1) lies within the non-coding exon 1 and generates transcripts to produce the complete N-terminal region of p53. These transcripts have the potential to drive the synthesis of four N-terminal variants through alternative translation initiation, as described in the next paragraph (75,76). In light of previous knowledge regarding *TP63* gene architecture and the presence of two independent promoters, a second promoter was searched for in *TP53*. Indeed, transcripts derived from a putative promoter located in intron 4 (analogous to exon 3′ of *TP63*) were detected using highly sensitive nested PCR (70 cycles, approximately 10^20^-fold amplification) (77) (Figure 4). The mRNA derived from this promoter contains two in frame AUG codons at +396 and +495 in exon 5 (positions refer to the full-length p53 coding sequence) that drive translation initiation of two N-terminal isoforms, Δ133p53 and Δ160p53 (see below for details) (77,78). It is not clear from GTEx data that the P2 transcript is expressed in normal adult human tissues.

### Alternative initiation of translation

Initiation of translation at +1 of the P1 *TP53* mRNA gives rise to canonical p53, whilst initiation at +118 produces the p47 isoform (also known as Δ40p53α) that lacks the first 39 amino acids, including TA1 and Box-I (79,80) (Figure 4). Similarly, translation initiation at the first or second AUG in exon 5 of the P2 transcript (AUG codons at +396 and +495) would give rise to Δ133p53 and Δ160p53 isoforms (77,78) (Figure 4). These P2-derived proteins lack TA1, TA2, Box-I, the PRR and part of the DBD. Notably, the two AUGs in exon 5 are also present in the P1 transcript and, considering the seemingly low levels of mRNA derived from P2, it will be important to know if these protein isoforms arise from the full-length transcript. In theory, 3′ alternative variants could occur in the longer and shorter N-terminal isoforms (Figure 4).

### Evidence for p53 protein isoforms

The detection of proteins originating from different *TP53* transcripts and translation initiation sites is not well established. There are many well-characterized antibodies to p53 with known epitopes (81,82) that can be used in combination to detect the presence or absence of specific p53 domains by Western blotting, and bands can be putatively assigned as specific isoforms by their relative size in comparison to overexpressed recombinant p53 isoforms. Some polyclonal reagents have been produced against specific isoforms, but show questionable reactivity and results that do not correlate with mRNA analyses (83,84), which in any case should be considered with caution due to the abundant post-transcriptional control observed for p53 [reviewed in (55,85–88)]. Therefore, improved reagents including mono-specific antibodies to individual p53 isoforms will be required to identify p53 protein isoforms definitively.

An indirect approach to estimate which transcripts give rise to proteins is the detection of mRNAs present on isolated mature ribosomes (heavy polysomes). Conversely, mRNAs associated with free ribosomes (light fractions) are not translated with high efficiency (89,90). As an example, in a pilot experiment, we found that 98% of *TP53* P1 transcripts in proliferating HEK293 cells are located in heavy polysomes (i.e. active protein synthesis fraction), whereas transcripts from the *TP53* P2 promoter are found mainly in fractions containing free ribosomal subunits. Combined with the relative paucity of P2-derived transcripts, their lack of accumulation in heavy polysomes indicates that they are not only rare, but also less actively translated. Similarly, the level of *TP53β* transcripts associated with heavy polysome fractions is less than 1%, while *TP53γ* mRNAs are absent from these actively translating fractions (Figure 5B). These observations together with GTEx data suggest that mRNAs coding for C-terminal p53 variants are relatively minor forms and appear to be less actively translated compared to *TP53α*. We stress that this example is from only one cell line under normal growth conditions and there may be differences in the translation of transcripts depending on cell type and conditions.

The alternatively initiated p47 isoform (Δ40p53α) was the first p53 isoform detected in non-transformed and cancer cell lines from diverse origins (79,80). Because this isoform does not contain unique amino acids at the N-terminus, p47 isoform-specific antibodies are difficult to produce and the only polyclonal antibody raised so far (ACMDD) also cross-reacts with full-length p53 (91). Despite the lack of p47-specific reagents, Western blotting with an antibody exclusion approach suggested that p47 is synthesized in primary human neural progenitor and pluripotent embryonic stem cells, as well as glioblastoma (92,93).

Detection of alternatively initiated Δ133p53 and Δ160p53 face similar difficulties. A polyclonal serum (MAP4) raised against the free N-terminus of Δ133p53 detects Δ133p53α following expression of a cDNA lacking the first 132 codons of p53 (94). A protein with a size corresponding to Δ133p53α is reported in some human tissues and primary cultures, in cancer samples and cell lines (78,94–100). Moreover, Δ133p53α has been suggested to be accompanied by Δ160p53α in some cancer cell lines, but this isoform has not yet been detected in normal tissues or cancer samples (78,96,100).

Proteins from translation of the cryptic exon 9β-retaining transcripts can be detected using polyclonal sera (KJC8 and TLQ40) raised against the unique C-terminal peptide sequence. These antibodies detect a protein corresponding to the size of p53β in cell lines (77,101–103), normal human tissues, primary cultures (94,98) and cancer samples (94,97,103). The other putative β forms derived from P1 and P2 transcripts are suggested to be produced only in cell lines (77,78,94,103). However, due to the lack of specificity of the polyclonal antibodies, which recognize numerous bands by Western blotting, and the similar apparent size of some p53 isoforms and the low frequency of P2 transcripts, it is difficult to assign individual bands definitively to specific variants.

In line with the lack of *TP53γ* mRNAs in GTEx, there is little evidence for proteins derived from these transcripts. However, interfering with mRNA splicing in cultured cells showed increased detection of a band of the approximate size of p53γ (47–48 kDa) using monoclonal and polyclonal p53 antibodies that recognize different epitopes within p53 (60). Again, this is not conclusive evidence but may instead represent differential p53 modification/proteolysis under the experimental conditions tested. On the other hand, that splicing inhibition induces *TP53γ* supports the notion that this alternative splice variant in cancer (27,70,71) may occur due to low splicing fidelity in tumour cells.

### Regulation and function of TP53 isoforms

Considering the importance of p53 in controlling cellular functions, transcription of p53 isoforms is expected to be tightly regulated, but there is little information on cellular conditions and molecular mechanisms that control p53 isoform expression. Evidence for mechanisms that control the choice of promoter usage or that regulate alternative splicing of *TP53* mRNAs is sparse. Reciprocal changes in *Δ133TP53α* and *TP53β* transcripts have been linked to fibroblast passage number and other senescence conditions, but the mechanisms are not known (94,97–99). Alternative promoter usage during senescence may be regulated by epigenetic changes, and one study reported non-random demethylation events within *TP53* exon 5 during continued cell passage, with a similar pattern in adult compared to embryonic mouse tissues (104). However, this methylation profile does not affect transcription from P1 and P2, since their induction rate was similar in passaged and control cells (104). There are no reports of selective induction or repression of P2 over its P1 counterpart, which appears contradictory to the suggested downregulation of P2 promoter-encoded Δ133p53α reported to affect the onset of senescence in both cancer cell lines and primary cultures (78,94–100). However, as for the p47 isoform derived from the main *TP53* transcript (80,105–108), post-transcriptional regulatory mechanisms may control Δ133p53α protein synthesis *via* alternative initiation of translation, although there is no direct evidence for this.

Δ133p53α lacks the N-terminal TA domains but retains the OD, and it is possible that the replicative phenotype described above depends on interactions with p53 (and see below for p47). Whilst the levels of Δ133p53α are orders of magnitude lower than full-length p53, N-terminal truncated variants may be expressed preferentially in individual cells to control replicative cellular states and senescence. Thus, investigations of such intercellular heterogeneity will require single cell analyses at the protein and/or RNA level.

The p47 (Δ40p53α) variant arises from alternative initiation of translation following endoplasmic reticulum (ER) stress and activation of the unfolded protein response in a PERK kinase-dependent fashion (105–109). The mechanism for selecting the translation initiation codon is *via* a conformational change in the *TP53* mRNA structure (110). Interestingly, human but not murine *TP53* mRNA shows this PERK-dependent conformational alteration and consequently only human cells induce p47 under ER stress. Overexpressing p47 in cells causes G2 cell cycle arrest by upregulating 14–3-3σ and suppressing p21^CDKN1A^ (105,109). p47 has no effect on G1 transition, which is instead regulated by full-length p53 (111,112), pointing to the specialization of these two isoforms to control the cell cycle progression under different conditions (105,109). Transgenic mice overexpressing the corresponding p44 variant show a premature ageing phenotype, with early death accompanied by changes in stem cell pluripotency. Importantly, the phenotype was not observed in mice lacking *Trp53* (113,114), indicating that the two isoforms act together and highlighting the role of translationally-initiated isoforms in expanding the functional repertoire of p53.

Contrary to p53, which is induced at the protein level after multiple stresses, p53β protein is not induced by actinomycin-D (77). This is surprising since the +1 AUG initiation site and the surrounding regions that are shared between *TP53α* and *β* mRNAs enhance p53α translation following DNA damage (77,107,108,115). p53β also lacks the C-terminal ubiquitination lysine residues (370, 372, 373, 381, 382 and 386) that mediate MDM2-dependent degradation, thus its turnover rate is not affected by MDM2 that acts in a feedback loop to reduce p53α protein levels (63,116). These observations can be explained by the nonsense-mediated RNA degradation pathway of *TP53β* mRNA (62,63). Detailed descriptions of p53β function under stresses linked to DNA metabolism, as well as the molecular mechanisms responsible for controlling its expression, remain to be addressed.

### TP73

**Figure 6.**
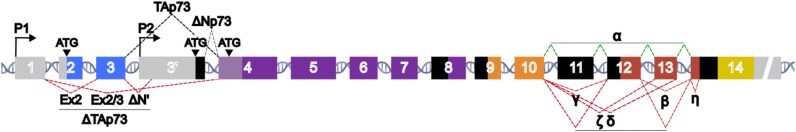
*Gene architecture of the human TP73 locus*. Boxes represent canonical and alternative exons and DNA cartoon represents introns. Exons are numbered according to canonical annotation and cryptic exon is denoted as 3′. Promoters P1 and P2 are represented by arrows and translation initiation codons (ATG) with arrowheads. Splicing patterns are annotated by dashed lines that connect the regions involved. Splicing at the 5' end (on the left) above the cartoon refers to splicing patterns resulting from alternative promoter usage, and below the cartoon refers to alternative splicing events. At the 3' end (on the right), canonical splicing is indicated above and alternative splicing patterns are indicated below. Shaded areas denote nucleotide sequences that correspond to different protein domains. Note that ΔTAp73 may originate in at least five ways; as a result of alternative splicing of Ex2, Ex2/3 and ΔN’, through use of P2 to transcribe exon 3′, as well as alternative translation initiation at an AUG located in exon 4.

The *TP73* gene *(Trp73* in mouse) was first discovered in 1997 and is located on chromosome 1p36.33 and organized into 14 canonical exons (117) (Figure 6). *Trp73* knockout mice show abnormal neural development, chronic inflammation and reduced fertility, but not tumour susceptibility (118). These abnormalities were originally ascribed to the direct effects of p73 loss in cells of the abnormal tissues, but can also be explained by indirect effects relating to the role of TAp73 as an inducer of multiciliated epithelial cells, which are required for cerebrospinal fluid and airway mucus flow (119,120). The p73 DBD shows strong similarity to p53, and a weak p53-like activity is observed in cells in which TAp73 isoforms have been overexpressed. Despite this ability, p73 is rarely mutated but is often overexpressed in specific human tumour types (121), presumably as ΔTAp73 isoforms (i.e. proteins that lack the TA domain; see below). The tissue distribution of ΔTAp73 forms and their physiological roles are unclear, but they are unlikely to relate to p53, which does not oligomerize with p73, but may involve influencing p63, which forms stable heterotetramers with p73 (122).

### Alternative splicing to produce N- and C-terminal variant p73 proteins

**Figure 7.**
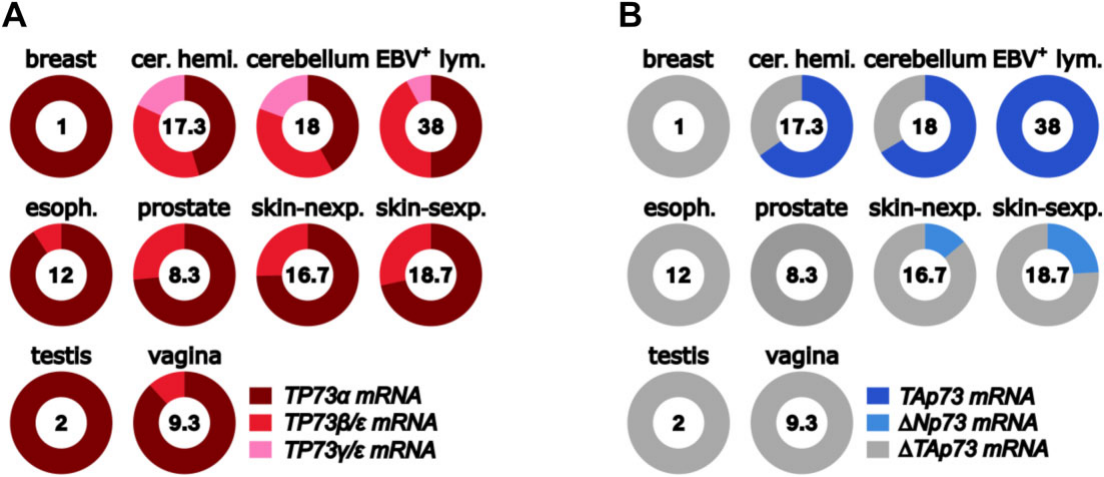
*The distribution of TP73 transcripts in human tissues*. (**A)** Proportions of alternatively spliced *TP73* mRNAs. Numbers inside the circles refer to the average number of reads for each tissue and isoform data represent the percentage of reads across 3′ exon–exon junctions that define the alternative splicing event, normalized according to the number of reads of non-spliced exon–exon junctions, as described in (33). *TP73α* cannot be determined from junction reads since there is no specific junction to define it. Therefore, values were estimated by subtracting the reads assigned to other isoform from the total number of reads. β and ϵ cannot be uniquely distinguished; thus, the values represent their sum. The same approach was used for γ/ϵ. (**B)** Proportions of *TAP73*, *ΔNP73* and *ΔTAP73* mRNAs. *ΔTAP73* levels were calculated as the number of *TP73* reads that are not assigned to *TAP73* or *ΔNP73* (*ΔEx2P73* and *ΔEx2P73* are undetectable). Note that absolute *TP73* mRNA levels vary considerably. Data are taken from GTEx. cer. hemi., cerebellar hemisphere; EBV^+^ lym., Epstein–Barr virus-positive lymphocytes; skin-nexp., skin not exposed to sun; skin-sexp., skin exposed to sun.

Two 3′ end splice variants (*TP73α* and *TP73β*) were identified during initial characterization, with *TAp73α* containing all 14 exons and *TP73β* lacking exon 13 (117). Since then, several other variant *TP73* transcripts have been proposed to result from alternative splicing at the 3′ end of the primary transcripts, providing seven different C-terminal isoforms (designated α, β, γ, δ, ϵ, ζ and η) (Figure 6) (123,124). Although these are annotated in Ensembl (Gene: TP73 ENSG00000078900), many are not well supported at the transcript level (Supplementary Table S3). These splice variant mRNAs were reported to show tissue-specific variation, suggesting that they possess distinct functions and regulation, but many isoforms rely on highly sensitive amplification methods for their detection (123,124). In GTEx, the most widespread and abundant form is *TP73α*, which is found in all tissues expressing *TP73* mRNA (Figure 7A and Supplementary Table S3). Indeed, *in situ* hybridization identified *TP73α* but not *TP73β*, *TP73γ* or *TP73*δ mRNAs during murine development (118).

In addition to 3′ alternative splicing, the 5′ end of P1-derived *TP73* mRNAs was reported to produce N-terminal truncated variants by single or double removal of exons 2 and 3 (ΔEx2p73 or ΔEx2/3p73) (Figure 6). These transcripts therefore produce proteins that lack the TA domain, collectively termed ΔTAp73 forms. Of these, splicing out of exon 2 (Δ*Ex2TP73*) was reported using PCR amplification followed by hybridization or by using radiolabelled PCR, and represents a minor form of *TP73* transcripts seen only in tumour cells (117,125,126). Removal of both exon 2 and exon 3 produces Δ*Ex2/3TP73* mRNA, a variant also seen only in tumours with highly sensitive detection methods (127). These transcripts are not seen in any tissue in GTEx. Another putative transcript that produces a ΔTAp73 protein is termed *ΔN’TP73*. This transcript is derived from P1, contains exons 1, 2 and 3, but exon 3 is spliced to a site within exon 3′ (128,129). Despite containing the canonical AUG of TAp73 within exon 2, this transcript is proposed to initiate translation from the AUG in exon 3′ to produce a protein identical to ΔNp73 (Figure 6). There is no evidence in GTEx for the presence of this splicing event in normal adult human tissues.

### Alternative promoters to produce N-terminal-truncated p73 proteins

*TP73* is proposed to contain two major promoters. The P1 promoter is located at exon 1 and drives mRNAs carrying the information to encode TAp73 proteins. By analogy with *TP63*, a potential second promoter was identified within intron 3, which transcribes exon 3′ to generate mRNAs coding for ΔNp73, lacking the p53/p63-like TA domain and containing a short unique amino acid sequence at the N-terminus (118,128,130), similar to the mechanism used to produce ΔNp63 (Figure 6). In addition, recent analyses identified a discrepancy between the amount of *TP73* exon 4 mRNA and the amounts of exon 3 and 3′ transcripts, which led to the discovery of a transcription start site immediately upstream of exon 4 (33). These transcripts would produce a ΔTAp73 protein from an in-frame initiating AUG codon in exon 4. GTEx does not include this proposal, but it is represented by the discrepant number of combined reads for *TAP73*, *ΔEx2P73*,*ΔEx2/3P73, ΔNP73* and *ΔN’P73* compared to the total number of *TP73* reads. Figure 7B therefore shows the relative proportions of *TP73* 5′ variants as *TAP73*, *ΔNP73* and the remainder labelled as *ΔTAP73* (*ΔEx2P73*,*ΔEx2/3P73* and *ΔN’P73* are not identified in any tissue).

Studies of human tumours have shown an increase in some 5′ splicing events in some tumours, but data are generally reported as fold-change rather than absolute values. Where available, quantitative data show that 5′ splice variants (*ΔEx2p73*,*ΔEx2/3p73, ΔNP73* and *ΔN’P73*) are minor forms in solid tumours compared to *TAP73* transcripts, which range from 10 to 10 000-fold higher levels than 5′ splice variants or alternative promoter derived transcripts in most tumours examined (131–133). In contrast, it has been reported that B-cell lymphomas show approximately 10-fold higher levels of *ΔNP73* than *TAP73* (134), which may relate to the disruption or deletion of chr 1p36 leading to decreased *TAP73* or increased *ΔNP73* mRNA (135).

### Alternative initiation of translation to produce N-terminal-truncated p73 proteins

The use of downstream AUGs for initiation of translation of *TP73* mRNAs was first proposed by the presence of a potential internal ribosome entry site adjacent to a strong Kozak consensus site within exon 4, representing the fifth in-frame AUG in *TAP73* mRNA (136). Support for the use of this site to initiate translation from *TAp73* mRNA came from transfected cells, with some evidence for regulation following DNA damage. This fifth AUG in *TAp73* mRNA is the same as the first AUG in transcripts derived from the recently discovered transcription start site immediately upstream of exon 4 that is proposed to be the major origin of ΔTAp73 isoforms (33).

### Evidence for p73 protein isoforms

There are many reports on p73 isoform transcripts, but p73 protein isoforms are not well documented due to a lack of reliable detection reagents. TAp73-specific antibodies are available that have been appropriately characterized (42), but most p73 antibodies are poorly characterized and often show cross-reaction with p63 and/or p53, or even non-specific binding to proteins outside the family (40,42). For example, although the mouse monoclonal antibody to ΔNp73 38C674.2 (also known as IMG-313A) has been widely used, it recognizes a non-specific protein band in Western blotting (42) and shows widespread nuclear and cytoplasmic staining in tumours (133,137). Some pan-p73 monoclonal antibodies with demonstrated specificity are available [e.g. (138)], and it is to be hoped that isoform-specific antibodies will be produced in the future. Additionally, the use of isoform-specific *in situ* hybridization is extremely sparse. Measurement of p73 transcripts from isolated polysomes would also help in identifying transcripts that are translated under different conditions.

### Regulation and function of TP73 isoforms

Unlike p53 and p63, no medical syndromes linked to *TP73* germline mutations are known, and the role as a human tumour suppressor or oncogene is not obvious as *TP73* is rarely mutated, lost or gained in cancer (121). Transgenic mice lacking exons 2 and 3 required for TAp73 synthesis (TAp73^−/−^ mice) are prone to spontaneous tumour formation, indicating a tumour suppressor activity for TAp73 (139), while targeting exon 3′ to produce ΔNp73^−/−^ mice indicates a major role in neural cell survival and choroid plexus development, with a reduced growth of transformed ΔNp73^−/−^ embryonic fibroblasts (140,141).

Apart from the different downstream pathways of p53 and p73 illustrated by the knockout animal phenotypes, upstream pathways are also different. The *TP73* P1 promoter is a target for induction by E2F1, Myc and E1A, and is repressed by C-EBPα and N-Myc (142–148). Methylation and mRNA stabilization have also been put forward as mechanisms to control *TAP73* and *ΔNP73* transcript production in leukaemias and other cancers (149–152). At the protein level, the WWP2 NEDD4-like E3 ubiquitin ligase controls TAp73 and a heterodimer of WWP1 and WWP2 controls ΔNp73 stability (153). ITCH is another p73 NEDD4-like E3 ubiquitin ligase that is downregulated after DNA damage, allowing accumulation of p73 (154). Several other PTMs, including phosphorylation and acetylation, have been implicated in regulating the stability and activity of p73 isoforms [reviewed in (155)].

In summary, the sources of N-terminal truncated (ΔTA) p73 variants are unclear at the present time and may result from alternative splicing of 5′ exons, alternative promoter usage and/or alternative AUGs used for translation initiation. Clearly, this is an important area for elucidation. Evidence for alternative C-terminal isoforms other than p73α is also insufficient in many instances and requires further investigation.

### Summary and perspectives

We have critically evaluated the evidence for isoforms in each member of the p53-family, highlighting the technical challenges to determine their origin, level of expression and regulation. Despite the data indicating that many alternative p53, p63 and p73 transcripts are rare in normal human adult tissues and tumours, it is important to remember that these observations do not rule out that there are specific conditions where they are present at sufficient levels to have an active physiological function, for instance, during embryogenesis, tissue differentiation, stress or ageing. Although isoforms in the p53-family undoubtedly have the potential to play important physiological roles, we conclude that more data are needed to understand how, or if, they play a part in differentiating the functional repertoire of their respective genes. These considerations have wider implications outside the p53-family, and standardized protocols for the detection and analysis of mRNA and protein isoforms would be beneficial for many genes/proteins.

Ideally, validation of isoforms should include a combination of assays: (i) detection and quantitation of steady-state levels and translation rate of mRNAs coding for different protein variants, both at cell population and single-cell scale; (ii) observation of protein isoforms with specific and sensitive antibodies or other reagents in both cell extracts and *in situ*; (iii) confirmation that their endogenous expression is affected when the specific coding mRNA is removed or altered in an appropriate physiological setting, with concomitant alterations to cellular behaviour; (iv) evidence for differential regulation of the isoform under specific conditions; and (v) evidence for the mechanism for differential regulation.

A recent database of splicing variations (156) can help in understanding tissue or cancer-specific mRNA splicing, while tools such as MANE, APRIS and TRIFID are reliable unbiased approaches to identify the functional relevance of alternative transcripts (157–159). Characterization of isoforms in the p53 family suffers from lack of reagents that can detect the encoded proteins and there is a clear need to develop isoform-specific reagents such as antibodies, scFv, monobodies or synthetic customizable protein binders such as DARPINs, affibodies or anticalins (160–164). Detection of peptides by mass spectrometry will also help but requires further improvement of current technologies to reach deeper proteome sequencing at the population or single-cell level (9,165). In particular, targeted N-terminal proteomics together with ribosomal profiling is useful for identifying translation-derived isoforms (166). Appropriate quantitative studies of mRNA and protein levels combined with careful knockdown of specific isoforms *in vivo* will be required to address these possibilities and confirm or refute the proposed roles of each isoform. For example, CRISPR-mediated deletion of *Trp63* exon 13 in mice resulted in higher p63β and lower p63α mRNA levels without affecting TA, ΔN or p63γ (167). These mice show female infertility due to depletion of oocytes that express TAp63, but no obvious effects on other tissues such as skin and thymus that express ΔNp63 protein variants. However, one needs to be cautious of this approach because cells now synthesize equal levels of p63α and p63β, altering the dominance of the targeted transcript from the normal physiological situation. The use of CRISPR-mediated insertion of tags would be an alternative method to track expression of individual isoforms *in vivo* or in cell lines to obviate the need for isoform-specific antibodies. A further issue that needs investigation regarding the functional properties of p53-family isoforms is their propensity to form mixed tetramers. Here, mis-sense p53 mutant proteins have been proposed to interact with p63 and/or p73 to provide gain of function activities (168). Many of these studies use overexpression and cell lysis followed by immunoprecipitation, potentially allowing protein complexes to form in solution. In contrast, endogenous p63–p73 interactions have been shown using DARPINs that bind to the heteromeric form only (122), and techniques such as proximity ligation assays (169) can also identify heteromeric p53/p63/p73 *in situ*, although identification of tetramers containing specific isoforms would require suitable reagents.

A common thread in the identification of p53-family isoforms has been that ‘if alternative promoters or splicing occurs in one gene in the family, they should also occur in the others’. Although this may be true and is common during gene evolution, it is a risky logic, and targeted searches for potential isoform sequences with highly sensitive detection methods will detect rare transcripts, with their importance potentially overemphasized. In addition, this line of reasoning assumes that the p53-family has evolved solely through divergent evolution, where the ancestral gene (*TP63* in this case) was duplicated twice to form the *TP73* and *TP53* genes, and these have retained the same gene architecture (i.e. two or more promoters and alternative 3′ exon splicing) but have diverged in nucleotide sequence to produce proteins with slightly different properties. An alternative hypothesis is similar to the concept of convergent evolution, where a useful property is not inherited directly but develops independently. As examples, enzymes with a slightly different but similar function may have evolved from a related enzyme (divergent evolution) or from an unrelated enzyme (convergent evolution) (170), different families of viruses have evolved diverse mechanisms of maturation through convergent evolution to achieve greater sophistication of this process (171), and the evolution of eyes and photoreceptors involved divergent and convergent evolution paths to produce the various eye-types seen in modern organisms (172). For the p53-family, our observations tend to support convergent evolution for producing N-terminal truncated proteins in each family member. Indeed, alternative promoter usage and the cryptic exon 3′ is clear for *TP63*, whilst the available evidence indicates that the corresponding exon 3′ in *TP73* is not the major source for N-terminal truncated p73 proteins, and the separate mechanism of alternative AUG usage is used to produce N-terminal p53 variants (for which there is conversely no evidence in p63). Thus, the production of N-terminal truncated proteins appears to have evolved through different and unrelated mechanisms, implying convergent gene evolution to produce the same useful property. A critical unanswered question here is when during species evolution did *TP63* develop two N-terminal isoforms driven by alternative promoters—was it before or after gene duplication events that led to *TP73* and *TP53*? Similarly, when did the ancestral gene evolve C-terminal isoforms—before or after gene duplication events? The increasing whole genome and particularly whole transcriptome sequencing of diverse species will help in answering these intriguing questions.

A second common theme in p53-family research, as in many other fields, is the use of functional studies employing overexpression of particular isoforms and then measuring the effects on gene expression or biological behaviour. Although overexpression approaches provide important information for structure–function studies (72), they are less valuable for demonstrating or understanding true physiological function; unless the levels of exogenous isoform are controlled to mimic a natural condition, such studies show what could happen, not what actually happens in a physiologically relevant situation. These considerations are pertinent for many p53-family isoform studies, and additional approaches are required to show which transcripts are present under specific conditions prior to investigating their potential function. For example, comparative RNA analysis will help in determining the relative amounts of different transcripts, where a simple Northern blot has the advantage of accurate comparison of relative transcript levels, and isoform-specific probes can identify specific sets of variants. RT-qPCR analysis of transcripts isolated from polysome fractions helps in minimizing the risks of detecting transcripts that are not actively translated. In combination with state-of-the-art long-range and single-cell sequencing and improved detection tools (173) and/or droplet digital PCR, these approaches will give a better idea of the presence and amounts of transcripts under different conditions.

In addition, current studies in cancer versus normal tissues suffer from an absence of controls; an increase/decrease in a particular splicing event does not indicate a deliberate intention by the cell to increase/decrease that specific isoform, but may instead be a marker of general splicing accuracy or the activity of a particular splicing-associated factor, which are known to be aberrant in cancer (73,74,174,175). This is relatively easy to control for by employing the same approach with the same level of sensitivity to examine splicing of one or more genes that have similar gene structures but no known role in carcinogenesis of that type. To our knowledge, this simple assessment has not been performed, leaving the significance of changes in p53-family isoforms unclear—are altered mRNA splicing patterns of p53-family members a cause or a consequence of the tumour biology?

Finally, a key aspect that is lacking for many putative isoforms is their mechanism of regulation. Isoforms that play different physiological roles should be tightly regulated to control their activity, yet this information is missing for most p53-family isoforms, including how one promoter is used preferentially over another and how alternative splicing is controlled to adapt to cellular requirements. With the low levels of many isoform transcripts, it is difficult to determine whether alternative splicing represents a purposeful cellular process to produce a particular isoform, or instead represents erroneous activities and is simply a passenger alteration. Thus, extensive effort is needed to shed light on the important aspect of isoform control, and once this level of information is available, it will be possible to generate animal models in which endogenous isoforms can be manipulated in a physiologically relevant manner to uncover their physiological effects and potential therapeutic value.

## Supplementary Material

gkae855_Supplemental_File

## Data Availability

Isoform expression-related datasets of normal adult human tissues were derived from the Genotype-Tissue Expression (GTEx) project portal (https://www.gtexportal.org/home, GTEx Analysis Release V8, dbGaP Accession phs000424.v8.p2 accessed on 01/02/2024 and 10/08/2024). The preliminary data related to ribosome profiling analysis will be shared on reasonable request to the corresponding authors.
