# Supplementary material for: Re-appraising the evidence for the source, regulation and function of p53-family isoforms
Source: Nucleic Acids Res. 2024 Oct 15;52(20):12112–29. doi: 10.1093/nar/gkae855 (PMC11551734; doi:10.1093/nar/gkae855)
Supplement: gkae855_Supplemental_File [file gkae855_supplemental_file.pdf]

Supplementary data

Supplementary Table 1. *TP63* isoforms listed in Ensembl.

Transcript ID	Ensembl	bp	Protein (aa)	Translation ID	UniProt	RefSeq Match	Name	TSL[1]
ENST00000264731.8	TP63-201	4944	680aa	ENSP00000264731.3	Q9H3D4-1	NM_003722.5	TAp63α	1
ENST00000354600.10	TP63-203	4677	586aa	ENSP00000346614.5	Q9H3D4-2	NM_001114980.2	ΔNp63α	1
ENST00000392460.7	TP63-204	1668	555aa	ENSP00000376253.3	Q9H3D4-3	-	TAp63β	1
ENST00000392463.6	TP63-206	1386	461aa	ENSP00000376256.2	Q9H3D4-4	-	ΔNp63β	1
ENST00000418709.6	TP63-207	2810	487aa	ENSP00000407144.2	Q9H3D4-5	-	TAp63γ	1
ENST00000437221.5	TP63-209	2500	393aa	ENSP00000392488.1	Q9H3D4-6		ΔNp63γ	1
ENST00000440651.6	TP63-202	2032	510aa	ENSP00000317510.5	Q9H3D4-7	-	TAp63δ	1
	TP63-205	1251	416aa	ENSP00000376254.3	Q9H3D4-8	-	ΔNp63δ	1
					Q9H3D4-9		TAp63ε	
ENST00000449992.5	TP63-211	1506	501aa	ENSP00000387839.1	Q9H3D4-10	-	ΔNp63ε	1
	TP63-210	2031	676aa	ENSP00000394337.2	Q9H3D4-11	-	Δ-ΔNp63 (ΔNp73L)	1
ENST00000456148.1	TP63-212	1749	582aa	ENSP00000389485.1	Q9H3D4-12	-	None (p63δ)	5
	TP63-208[2]	676	183aa	ENSP00000401661.1	C9JW72	-		5
ENST00000460036.1	TP63-213	1753		-	-	-		1
ENST00000486398.1	TP63-214	546		-	-	-		3

Data are from Ensembl (Gene: TP63 ENSG00000073282) accessed November 2023, plus additional common names used in the literature for the indicated isoforms. [1]TSL, Transcript Support Level; TSL1, A transcript where all splice junctions are supported by at least one non-suspect mRNA; TSL3 A transcript where the only support is from a single EST; TSL5, A transcript where no single transcript supports the model structure. [2]Computational only.

Supplementary Table 2. *TP53* isoforms listed in Ensembl.

Transcript ID	Ensembl	bp	Protein (aa)	Translation ID	UniProt	RefSeq Match	Name	TSL[1]
ENST00000269305.9	TP53-201	2512	393	ENSP00000269305.4	P04637-1	NM_000546.6	p53α	1
ENST00000359597.8	TP53-202	1152	343	ENSP00000352610.4	J3KP33	-	NR	1
ENST00000413465.6	TP53-203	1018	285	ENSP00000410739.2	E7EQX7	-	NR	1
ENST00000420246.6	TP53-204	2653	341	ENSP00000391127.2	P04637-2	-	p53β	1
ENST00000445888.6	TP53-205	2506	393	ENSP00000391478.2	P04637-1	-	p53α	1
ENST00000455263.6	TP53-206	2580	346	ENSP00000398846.2	P04637-3	-	p53γ	1
ENST00000503591.2	TP53-207	2552	393	ENSP00000426252.2	E9PCY9, K7PPA8	-	p53α	5
ENST00000504290.5	TP53-208	2331	214	ENSP00000484409.1	P04637-9	-	Δ133p53γ	1
ENST00000504937.5	TP53-209	2271	261	ENSP00000481179.1	P04637-7	-	Δ133p53α	1
ENST00000505014.5	TP53-210	1261	No protein	-	-	-	-	2
ENST00000508793.6	TP53-211	2660	393	ENSP00000424104.2	E7EMR6, K7PPA8	-	p53α	4
ENST00000509690.6	TP53-212	2106	261	ENSP00000425104.2	E7ESS1	-	Δ133p53α	4
ENST00000510385.5	TP53-213	2404	209	ENSP00000478499.1	P04637-8	-	Δ133p53β	1
ENST00000514944.6	TP53-214	2170	300	ENSP00000423862.2	E9PFT5	-	NR	1
ENST00000571370.2	TP53-215	2275	No protein	-	-	-	-	NA
ENST00000574684.1	TP53-216	104	No protein	-	-	-	-	1
ENST00000576024.1	TP53-217	175	31	ENSP00000458393.1	I3L0W9	-	NR	1
ENST00000604348.6	TP53-218	2488	386	ENSP00000473895.2	S4R334	-	NR	4
ENST00000610292.4	TP53-219	2639	354	ENSP00000478219.1	P04637-4	-	p47 (Δ40p53α)	1
ENST00000610538.4	TP53-220	2580	307	ENSP00000480868.1	P04637-6	-	Δ40p53γ	1
ENST00000610623.4	TP53-221	2331	187	ENSP00000477531.1	A0A087WT22	-	Δ160p53γ	1
ENST00000618944.4	TP53-222	2404	182	ENSP00000481401.1	A0A087WXZ1	-	Δ160p53β	1
ENST00000619186.4	TP53-223	2271	234	ENSP00000484375.1	A0A087X1Q1	-	Δ160p53α	1
ENST00000619485.4	TP53-224	2506	354	ENSP00000482537.1	P04637-4	-	p47 (Δ40p53α)	1
ENST00000620739.4	TP53-225	2579	354	ENSP00000481638.1	P04637-4	-	p47 (Δ40p53α)	1
ENST00000622645.4	TP53-226	2653	302	ENSP00000482222.1	P04637-5	-	Δ40p53β	1
ENST00000635293.1	TP53-227	1883	410	ENSP00000488924.1	A0A0U1RQC9	-	NR	5

Data are from Ensembl (Gene: TP53 ENSG00000141510) accessed November 2023. [1]TSL, Transcript Support Level; TSL1, A transcript where all splice junctions are supported by at least one non-suspect mRNA; TSL2, A transcript where the best supporting mRNA is flagged as suspect or the support is from multiple ESTs; TSL4, A transcript where the best supporting EST is flagged as suspect; TSL5, A transcript where no single transcript supports the model structure; TSL-NA, A transcript that was not analysed for TSL. NR: non-reported protein variants.

Supplementary Table 3. *TP73* isoforms listed in Ensembl.

Transcript ID	Ensembl	bp	Protein (aa)	Translation ID	UniProt	RefSeq Match	Name	TSL[1]
ENST00000346387.8	TP73-201	4854	540	ENSP00000340740.4	O15350-6	-		5
ENST00000354437.8	TP73-202	2140	499	ENSP00000346423.4	O15350-2	-		5
ENST00000357733.7	TP73-203	4899	555	ENSP00000350366.3	O15350-13	-		5
ENST00000378280.5	TP73-204	2062	426	ENSP00000367529.1	A0A0C4DFW9	-		1
ENST00000378285.5	TP73-205	2117	450	ENSP00000367534.1	O15350-9	-		1
ENST00000378288.8	TP73-206	5120	587	ENSP00000367537.4	O15350-8	-		1
ENST00000378290.4	TP73-207	1831	565	ENSP00000367539.4	O15350-11	-		2
ENST00000378295.9	TP73-208	5192	636	ENSP00000367545.4	O15350-1	NM_005427.4		1
ENST00000603362.5	TP73-209	1668	555	ENSP00000474626.1	O15350-13	-		5
ENST00000603364.5	TP73-210	1551	No protein	-	-	-		2
ENST00000604074.5	TP73-211	4806	403	ENSP00000475143.1	O15350-4	-		5
ENST00000604194.1	TP73-212	1092	No protein	-	-	-		1
ENST00000604479.5	TP73-213	1623	540	ENSP00000474322.1	O15350-6	-		5
ENST00000604566.1	TP73-214	1358	No protein	-	-	-		2

Data are from Ensembl (Gene: TP73 ENSG00000078900) accessed November 2023. [1]TSL, Transcript Support Level; TSL1, A transcript where all splice junctions are supported by at least one non-suspect mRNA; TSL2, A transcript where the best supporting mRNA is flagged as suspect or the support is from multiple ESTs; TSL5, A transcript where no single transcript supports the model structure.
